# From naturalistic neuroscience to modeling radical embodiment with narrative enactive systems

**DOI:** 10.3389/fnhum.2014.00794

**Published:** 2014-10-06

**Authors:** Pia Tikka, Mauri Ylermi Kaipainen

**Affiliations:** ^1^Department of Film, Television and Scenography, Aalto University School of Arts, Design and ArchitectureHelsinki, Finland; ^2^Department of Media Technology, Södertörn UniversityHuddinge, Sweden

**Keywords:** enactive systems, neuroimaging, narrative nowness, radical embodiment, naturalistic neuroscience, time experience, context dependency

## Abstract

Mainstream cognitive neuroscience has begun to accept the idea of *embodied mind*, which assumes that the human mind is fundamentally constituted by the dynamical interactions of the brain, body, and the environment. In today’s paradigm of naturalistic neurosciences, subjects are exposed to rich contexts, such as video sequences or entire films, under relatively controlled conditions, against which researchers can interpret changes in neural responses within a time window. However, from the point of view of* radical* embodied cognitive neuroscience, the increasing complexity alone will not suffice as the explanatory apparatus for dynamical embodiment and situatedness of the mind. We suggest that narrative enactive systems with dynamically adaptive content as stimuli, may serve better to account for the embodied mind engaged with the surrounding world. Among the ensuing challenges for neuroimaging studies is how to interpret brain data against broad temporal contexts of previous experiences that condition the unfolding experience of *nowness*. We propose means to tackle this issue, as well as ways to limit the exponentially growing combinatoria of narrative paths to a controllable number.

## Introduction

The embodied mind view assumes that the phenomenon of human mind is fundamentally constituted by the dynamical interactions of the brain, body, and its environment (Merleau-Ponty, 1962; Varela et al., 1991; Clark, 1997; Chemero, 2009; Barrett, 2011). In this article, we first lay out the path we see leading from conventional to naturalistic neuroscience. Then we discuss what we envision as the next steps on the way toward radically embodied cognitive neuroscience.

## Conventional to naturalistic neurosciences

Cognitive neurosciences following the conventional experimental paradigms of psychology and psychophysiology have typically departed from simple stimuli that are *isolated* from bodily and situational contexts. The underlying epistemology is close to that of classical empiricism, with the aim to minimize the number of variables in order to make inductive inferences. This approach is, by definition, unable to handle complex systems of contexts as explanations of observations. Unlike in psychology, where the requirement of ecological validity has been long since recognized (Brunswik, 1955), the limits of computational capabilities have heavily constrained the possibilities of ecologically valid neuroscience. Recently, however, the advanced technological developments have allowed the adoption of a more *naturalistic* approach. The increasing use of stimuli, whose richness of characteristics and contextual embeddings assumedly corresponds to natural settings, marks a major methodological revision in neuroscience (see e.g., Hari and Kujala, 2009; Zaki and Ochsner, 2009; Maguire, 2012). Instead of experimental designs with isolated stimuli, the paradigm of *naturalistic neurosciences* is focused on the human cognition as it is engaged in more ecologically valid settings of longer durations, with the purpose to gain access to the brain processes that unfold in time.

So far, the method of free film viewing in the fMRI scanner has allowed observing human brain functions in relation to contexts reminiscent to “real-life” situations (Bartels and Zeki, 2004; Hasson et al., 2004; Malinen et al., 2007). The interpretation of the brain responses to film stimuli is typically based on annotation of the features and content factors of interest in the film at particular time points (Lahnakoski et al., 2012). The advantages of free film viewing are many. Films are, on one hand, highly controllable and can be repeated identically to a large number of people, and, on the other, often tell stories embedded in contexts of everyday experience. The use of naturalistic narrative stimuli in fMRI has already accumulated, for instance, the knowledge on the multiplicity of temporal hierarchies in the brain that may range from milliseconds to tens of seconds (Hasson et al., 2008; Kauppi et al., 2010). These findings allow relating neural behavior in different time scales to temporally extended contextual (or situational) factors, such as narrative comprehension, unfolding in time (Lerner et al., 2011). Further, the naturalistic settings allow studying intersubjectively shared aspects of cognition, such as contextual framing effects on social attributions (Mobbs et al., 2006), or emotional contagion (Nummenmaa et al., 2012), that reveal dependency of experience on contexts and perspectives in social settings.

**Figure 1:**
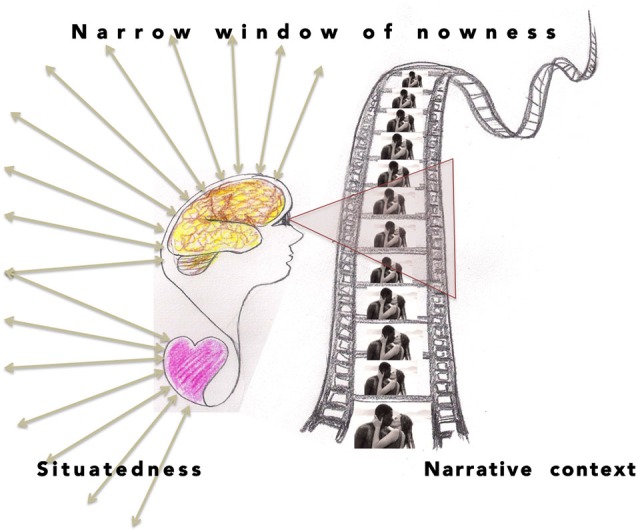
**In naturalistic brain experiments with film footage as stimuli, the observed brain activity is typically analyzed against the narrow window of nowness that is accessible through whatever is more or less immediately present, while ignoring the broader temporal contexts, for instance, the experiential history or situatedness of the subject, or the contextual effects of previous events on the comprehension of the immediate present**.

While movies have turned out to serve the purposes of naturalistic neuroscience to some point, they have major shortcomings with respect to radical embodiment. First, though resembling real-life situations, stimulus movies are typically not interactive, and thus not dynamic in the strict ecological sense. Therefore they cannot fully capture brain responses associated with sensorimotor and social enactment in similar manner as they would be elicited in real-life interaction situations. Secondly, movie stimuli give only limited access to extended temporal contexts of situations against which the cinematic story is experienced at a given transient moment of time. Brain activity is often analyzed through a narrow temporal window capturing not much more than that which is more or less immediately audiovisually present in the narrative content (Figure 1), whereby the analysis runs the risk of missing the subject’s temporally laid out engagement and experiential history conditioning the experience of that which is present.

Relevant questions related to studying human mind in socially valid contexts include, to what extent does the brain activity of “living-by” other people’s experiences on the screen differ from that of direct interaction between people? As an example from game studies, Kätsyri et al. (2013) have demonstrated the substantial qualitative difference between playing and just watching, motivating the goal of deeper engagement in neuroimaging studies. Currently, methods are being developed for scanning synchronous brain activations of two people who are in direct interaction in magnetoencephalography (MEG) with one another via real-time video (Baess et al., 2012), as well as in direct bodily interaction in a two-person fMRI scanner (Lee et al., 2012). Future will show, how two-person neuroimaging will meet the challenges of making analytical inferences of a system consisting of two “black boxes” instead of one. Meanwhile, in our view, the methodological gap between the one-person movie-viewing to the two-person interaction settings could be bridged with developing dynamically adaptive stimulus systems for managing socially valid interaction settings in more controllable manner. We propose that the explanatory framework of *enactment* might provide ways for modeling computationally the systemic situatedness of the brain and applying these means as dynamically adaptive stimuli. However, there is no reason to underestimate the methodical and epistemological challenges that will be touched in the following discussion.

## Enactive neuroscience

The notion of enactive cognitive science, as proposed by Varela et al. (1991), refers to the assumption of the mind as a brain-body system inseparably embedded in the world in terms of *enactment*, i.e., active engagement within that world, and that “cognition is not the representation of a pregiven world by a pregiven mind but is rather the enactment of a world and a mind on the basis of a history of the variety of the actions that a being in the world performs” (p. 9). In their terms, enaction consists of two points: (1) perception as perceptually guided action; and (2) the emergence of cognitive structures “from the recurrent sensorimotor patterns that enable action to be perceptually guided” (Ibid., p. 173). Applied to neuroscience, the radical enactive approach, as it is termed, should imply modeling the reciprocal relationship between embodied cognitive states and the corresponding neuronal activity at the level of large-scale emergent dynamical patterns of activity that cut across the brain, body, and the world (Thompson and Varela, 2001).

In order to fully accommodate enactment in the world, enactive stimuli should not be limited to static representations of the world, but rather to be extended to interactive stimulus models that are adaptive and capable of modeling the reciprocal impact of subject’s enactions to themselves. One possible way is related to the *interactive brain hypothesis* of Di Paolo and De Jaegher (2012) that suggests self-organizing dynamics of social engagement constituted by *states of coordination*, and* transitions between* the states. While using real persons or natural(-like) situations as stimuli may elicit “natural” brain activity, the downside is that the stimulus may remain as another “black box”, this is, as unpredictable and difficult to read and control as the brain. Another is to model the dynamics of relevant socio-cultural exchange (Kaipainen, 1994; Kitayama and Park, 2010), which, however, involves assumptions about cultural dynamics beyond the present focus.

In the following, we discuss the possibility of using narration-based dynamical simulations, such that are able to reflect relevant aspects of the mind’s situatedness and embodiedness in a similar manner as movies do, but which, in addition, are interactive and dynamic while maintaining a certain level of controllability.

## Narrative enactive system

Our proposition to bridge radical embodied cognitive views with naturalistic neuroscience draws from the concept of *narrative* *enactive* systems (Tikka, 2008; Kaipainen et al., 2011). It implies the application of dynamically generated or composited naturalistic stimuli for neuroscientific experiments (Tikka et al., 2012) as dynamical models of the live situations, in which the experiencer participates and acts in the framework of a story. Rather than fixed stimulus representation, such as linear movie footage, narrative enactive systems simulate the dynamically changing flow of the environment that is adaptive to the viewer’s bodily experience (perception and action-simulation) of the unfolding narrative. We envision that enactive narrative stimuli could be composited in real-time by a means of, for example, re-combinations of audiovisual media clips from a database (Tikka et al., 2006; Pugliese et al., 2014), algorithmically implemented animations, or game-like avatars with the model described above. This arrangement would constitute an *enactive medium*, capable of simulating life-like situations, in which the subject is engaged as a participant rather than just an observer.

**Figure 2:**
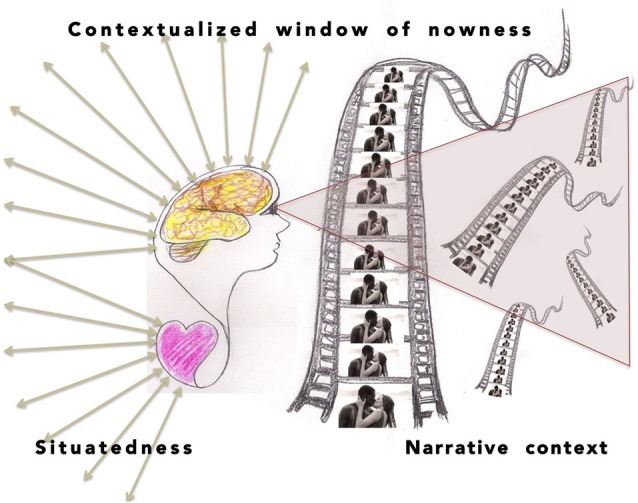
**The viewer has a contextualized and situated window of nowness to several levels of narrative, including retentive references to earlier events and eliciting protentive expectations for the coming events**. The temporal contexts can be weighted or prioritized in a number of ways, amounting to different interpretations (perspectives) of the narrative. They correspond to different narrative sequences that can be algorithmically composited.

Unlike traditional neuroscience paradigms, including the one referred to as “naturalistic”, an enactive feedback loop is assumed in the proposed setting. That means taking into account the contexts constituted by experiences of the previous events, implicitly guiding every experience of *nowness* (Figure 2). We assume that the experience of nowness is related to ongoing processes of memory and attention, corresponding to that which Husserl and later Varela have called *retention* (Husserl, 1964; Varela, 1999; Figure 2). Following their conceptualization, we take that retention is carried along as backward-looking reflections of the continuous process of enactment.

In order to substantiate this idea, imagine thriller film footage that shows a bedside drawer in a hotel room. In this situation, the mere presence of the piece of furniture on the transient screen is not enough to account for the excitement elicited in the viewer that has seen the movie from the beginning. It is essential to include the retentive context of having learned about the gun hidden there in a preceding stage of the narrative. This, in turn, implies the anticipation of a violent event, in Husserlian terms *protention*, likely to elicit certain kinds of brain responses reflecting alertness and readiness for motor action.

The assumption of enactive media (Kaipainen et al., 2011) implies that the experience itself (involving perceptual and action-related neural networks) steers the narrative via some kind of psychophysiological interface, e.g., eye tracking, electrodermal conductivity, or real-time neurofeedback, reflecting aspects of the holistic experience. At the same time, even the stimulus narrative itself has its own forward-driving inertia, corresponding to how environmental events flow independently of the observer, motivating anticipations (protentions). The methodological advantage is that both the history of such enactions (retention) as well as time-coded annotations of the narrative is known. These can be further aggregated into a time-dependent simulation of the *experiential perspective* (nowness) at each particular point of stimulus presentation, eventually providing a spectrum of cues for interpreting corresponding brain activity beyond the mere immediate stimulus. A preliminary computational model that simulates such an evolving experiential perspective, termed *nowness model*, has been introduced by Kauttonen et al. (2014). It consists of (a) decaying memory traces of previous experiences; and (b) a mechanism of refreshing traces via emerging associations. The nowness model, unlike conventional stimuli of neuroimaging studies, would be able to stand for a spectrum of contextualizing narrative factors, such as the hidden gun (referring to the previous example).

A practical challenge is obviously that the combinatoria of narrative paths may well grow too broad to allow sufficient statistical momentum for brain imaging studies, unless constrained to some manageable number. Our preliminary suggestion is to limit the variations to a controllable number of prototypical narrative categories, such a reduction might follow, for example, studies of viewer responses related to different film genres (Grodal, 1997; Visch and Tan, 2009), or player-responses in interactive games (Mathiak and Weber, 2006; Kätsyri et al., 2013).

## Discussion

For neurosciences, the implication of the radical embodied cognitive approach is to move toward holistic and systemic study of the brain-world-body dynamics. We propose rethinking the role of stimuli in neuroscience: instead of static representations, should be conceived of as continuously evolving dynamical systems that are capable of connecting the past and future experiences and of accounting for the mutual influences between brain-body and its environment. In our view, Varela’s concept of enactment encapsulates the essence of this relation and allows ways to go beyond today’s naturalistic neuroscience.

Obviously, the proposed approach sets significant challenges for the methods of empirical neurosciences. However, while the complete solution would amount to nothing less than modeling the world around the brain, we believe it possible to isolate partial, focused and controllable dynamic stimulus systems to address essential aspects of radical cognitive neuroscience. This prerequires rich annotations that do not have to be limited to conscious, linguistic, and semantic aspects of the experience (e.g., researcher’s or community’s tags on the stimulus material), but can also include psychophysiological recordings associated with the material (psychophysiological annotations, e.g., heart rate, eye-movements, electrodermal conductivity), environmental conditions (e.g., light, loudness, temperature), and references to cultural contexts (e.g., genre, reviews, background information). Besides rich annotations, the approach requires using the simulation of how previous embodied experiences are present in the experience of newness. Binding together the past and the anticipation of future beyond mere point-like fixed stimuli would allow a temporally situated interpretation of the corresponding observations of brain activity.

Fully conscious of the complexity of translating the proposed approach to actual laboratory work, we are confident that the idea of stimuli that are dynamically adaptive to embodied actions can open new avenues to radical embodied cognitive neuroscience.

## Conflict of interest statement

The authors declare that the research was conducted in the absence of any commercial or financial relationships that could be construed as a potential conflict of interest.
